# The Fabrication and Bonding of Thermoplastic Microfluidics: A Review

**DOI:** 10.3390/ma15186478

**Published:** 2022-09-18

**Authors:** Amid Shakeri, Shadman Khan, Noor Abu Jarad, Tohid F. Didar

**Affiliations:** 1 Department of Mechanical Engineering, McMaster University, 1280 Main Street West, Hamilton, ON L8S 4L7, Canada; 2 School of Biomedical Engineering, McMaster University, 1280 Main Street West, Hamilton, ON L8S 4L8, Canada

**Keywords:** microfluidics, thermoplastics, fabrication strategies, bonding strategies

## Abstract

Various fields within biomedical engineering have been afforded rapid scientific advancement through the incorporation of microfluidics. As literature surrounding biological systems become more comprehensive and many microfluidic platforms show potential for commercialization, the development of representative fluidic systems has become more intricate. This has brought increased scrutiny of the material properties of microfluidic substrates. Thermoplastics have been highlighted as a promising material, given their material adaptability and commercial compatibility. This review provides a comprehensive discussion surrounding recent developments pertaining to thermoplastic microfluidic device fabrication. Existing and emerging approaches related to both microchannel fabrication and device assembly are highlighted, with consideration toward how specific approaches induce physical and/or chemical properties that are optimally suited for relevant real-world applications.

## 1. Introduction

Since their inception three decades ago, microfluidic platforms have repeatedly redefined state-of-the-art approaches to fluidic automation, biochemical assays, high-throughput clinical screening, and a plethora of other areas [1,2,3,4,5,6]. Glass and polydimethylsiloxane (PDMS) have represented two key materials of choice in the microfluidics space, due to their physical and chemical versatility. The use of both these materials in microfluidics has been comprehensively discussed in our recent reviews [7,8]. However, the widespread implementation of devices composed of glass and PDMS has been severely limited by their costly and laborsome fabrication process, poor scalability, and high variability between devices. From a materials perspective, PDMS microfluidic platforms have demonstrated poor surface treatment stability and high susceptibility toward biomolecule adsorption, making them impractical for most biomedical applications [9]. Furthermore, leaching of uncrosslinked oligomers of PDMS could be problematic for cell culture applications [10]. On a more macro-scale, these devices often malfunction under high-pressure conditions, making many post-fabrication processing strategies unfeasible. Recent research efforts have thus been directed toward the material optimization of microfluidic devices. To this end, thermoplastic microfluidic platforms have garnered significant interest, given their excellent material properties and low device cost, relative to their glass and PDMS device counterparts. Materials such as poly(methyl methacrylate) (PMMA), cyclic Olefin polymer (COP), cyclic olefin copolymer (COC), polystyrene (PS), polycarbonate (PC), polyethylene glycol (PEG) and polyester terephthalate (PETE) are considered cost-effective thermoplastic polymers for microfluidic fabrications, providing biocompatibility, gas permeability, and good optical properties [11,12,13,14].

There are two steps involved in the development of thermoplastic microfluidic platforms: (i) device assembly, that involves making the microfluidic channels and bonding the layers, and (ii) channel functionalization for the desired application—the latter of which we have extensively reviewed in a recent review paper [11]. In this review, we provide a comprehensive overview of recent developments in the thermoplastic device fabrication space. More specifically, we first provide a thorough account of the instrumentation and approaches that can be engaged to form microchannel networks within thermoplastic polymer substrates. We subsequently detail the different approaches for bonding these microchannels, with consideration towards channel functionalization, which often occurs in parallel. While different approaches for fabrication of microfluidics have already been discussed in some review papers [12,15,16], we intend to compare only those that are applicable to thermoplastic-based microfluidics and that have drawn a lot of attention in recent years for the aforementioned reasons. Furthermore, we review the efficacy of each method by indicating its associated benefits and shortcomings in comparison to other strategies, and possible improvements that could be made for microchannel forming and bonding techniques. By providing many examples for each strategy, one should be able to choose the right fabrication and bonding approach based on their polymer of interest and their requirements.

Given the active nature of thermoplastic microfluidic research, legacy approaches are constantly being modified to improve performance and commercial potential. This, paired with emerging device fabrication strategies, provides a research landscape in need of such an up-to-date source for thermoplastic device fabrication. As such, this review pairs foundational studies with recent works and emerging approaches to provide new insights into the direction of this field.

## 2. Forming the Microchannel Geometry

### 2.1. Hot Embossing

Hot embossing is a common technique for the mass production of thermoplastic polymers with medium costs [17,18,19,20,21,22,23]. In hot embossing, the polymer plate is heated above the glass transition temperature (Tg) while it is pressed against a master mold with channel protrusions by a hydraulic press to form the microfluidic arrays as cavities in the polymer. The thermoplastic polymer plates used in hot embossing could be fabricated by injection molding [24,25]. Before performing the hot embossing process, the polymer plate may be annealed to reduce residual stress [24]. Depending on the polymer type, thickness, polymer chain orientation, and the experimental design, the applied pressure and temperature vary. After the process, which takes a few minutes (e.g., 2–20 min), the pressure is usually maintained as the samples cools down to enhance the uniformity [20,26]. 

Hot embossing was done at 150 °C on a COC pellet for 6 min under 1.38 MPa, followed by maintaining the polymer for 10 min at 25 °C under the same pressure [26]. Additionally, PC microchannels were prepared via compressing PC plates on a photolithographic patterned silicon mold at 155 °C with 1.2 MPa for 2 min followed by 5 min at 50 °C, while the pressure was kept constant [17]. Young et al. hot embossed PS substrates against an epoxy mold at 125 °C under the 900 kgf pressure for 15 min [21]. COP/COC have shown superior performance compared to PMMA chips fabricated via hot embossing, with higher signal to noise ratios and higher electrophoresis efficiencies due to their low impurity levels and high glass transition temperatures [27]. The embossing reference temperature (143 °C) for COC/COP microchannels is determined by the viscoelastic property of these polymers, while other processing parameters, such as the temperature, time, and pressure in the cooling and demolding stages, are determined by the Taguchi method. A COP microfluidic channel is said to have a high repeatability and low substrate deformation when it exhibits the following optimized parameters: reference temperature 143 °C, holding time 2 min, pressure 1.6 MPa, and demolding temperature 80 °C [28].

**Figure 1:**
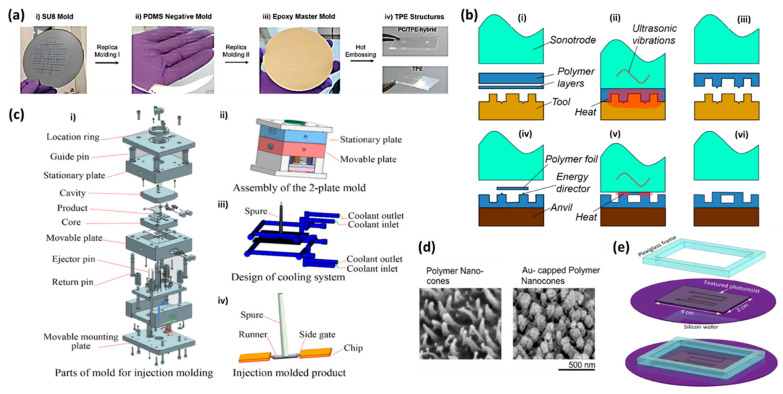
(**a**) Fabrication process of PC/TPE-hybrid microchannels via hot embossing method. Reproduced with permission [31] 2022, MDPI. (**b**) (**i**–**iii**) Incorporation of ultrasonic vibration in hot embossing process to form PC microchannels (**iv**–**vi**) ultrasonic welding of PC layers. Reproduced with permission [33], 2022, Springer. (**c**) Injection mold design for fabrication of PMMA microfluidic devices. (**i**) Injection molding parts, (**ii**) Mold assembly, (**iii**) Cooling system, (**iv**) Final product. Reproduced with permission [45] 2022, Springer. (**d**) SEM images of nanocones made of COC through injection molding techniques. Reproduced with permission [48] 2022, ACS. (**e**) Silicon mold with photoresist patterns and plexiglass frame. The setup was used to cast PDMS master molds for hot embossing COC and creating microchannels. Reproduced with permission [26] 2022, Elsevier.

Hot embossing is also a popular method to create microchannels out of thermoplastic elastomers (TPEs). The produced microchannels are transparent, flexible, and biocompatible [29,30,31]. Schneider et al. used this technique to make PC/TPE-hybrid microfluidic channels using epoxy-based master molds (Figure 1a) [31]. Hot embossing can be integrated with roll-to-roll printing, where a rotating embossing cylinder is used to transfer the microchannel features of the cylinder into a heated polymer web continuously fed into the system [32]. In order to create the embossing cylinder with the desired features, an embossing shim (thin strip of material on the cylinder) can be fabricated from a flexible steel using wet-etching and then laser welded to the cylindrical sleeve [32]. As roll-to-roll hot embossing continues heating and forming the substrate, it is considered a faster approach compared to normal hot embossing or micro-injection molding techniques. Runge et al. presented a different type of hot embossing process, where a PC thermoplastic polymer was pressed against a master mold via a tool capable of generating ultrasonic vibrations (sonotrode) (Figure 1b) [33]. The induced friction as a result of the vibrations could rapidly increase the temperature of the substrate above the glass transition temperature and form the desired patterns on the sheet. The process could be completed in a few seconds, which enables rapid replicating of thermoplastic microfluidics. However, the size of the channel that is possible to form by this method is typically limited to 50 µm to 1 mm in depth and 100 µm to 3 mm in width.

### 2.2. Injection Molding

Injection molding is another method frequently used to create microchannels in thermoplastics [25,34,35,36]. In this process, the thermoplastic polymer granules are melted (plasticization step) and injected into the mold cavity. The molten polymer is then solidified as the temperature decreases below the glass transition temperature (cooling stage) and finally ejected from the mold. The molding process is done under constant pressure to compensate for the shrinkage of the polymer during solidification. The process cycle takes seconds to a few minutes [35]. In addition to polymeric material properties, several process parameters, such as melt temperature and mold temperature, speed of filling and packing time as well as packing and holding pressures, attribute to the efficiency of the process and quality of the final product [37]. Ogorodnyk et al. have conducted a comprehensive review on the implementation of artificial intelligence (AI) methods for the monitoring and controlling of the parameters involved in injection molding [38]. The cooling stage is the most time-consuming part of the injection molding. In order to accelerate the process, rapid heating and cooling technologies by means of conformal cooling or variotherm system have been introduced. Conformal cooling could be conducted via accommodating cooling channels in the mold and conforming them to the shape of the mold cavity. The cooling channels can be made in different designs, such as spiral conformal cooling channels [39], milled grooved square shape conformal cooling channels [40], and longitudinal conformal cooling channels [41]. Conformal cooling significantly reduces the cooling time in a more uniform and consistent way by increasing the heat transfer efficiency, thereby enhancing the quality of the formed thermoplastic polymer. The channels in conformal cooling could also be used for heating the injected thermoplastic in order to prevent it from early solidification during the injection process as premature solidification can lead to defect formation in the product. In variotherm injection molding, the mold temperature is dynamically controlled according to each stage of the process. Before the injection, the temperature is raised to the glass transition temperature of the thermoplastic polymer. Then, the temperature is increased above the glass transition and kept constant during the mold filling step. Afterwards, the mold is rapidly cooled for the solidification of the polymer and the ejection step. Controlling the temperature could be performed by electromagnetic induction heating, which can heat the mold from 110 °C to 200 °C in only 4 s [42]. By using a proper coolant, the cooling time also takes only 20 s to reach 110 °C again [42]. Another way to heat the mold is to use steam at a temperature of 180 °C, which can increase the temperature in injection molding from 30 °C to 140 °C in 20 s [43]. Water can be used in this approach to cool the mold and solidify the injected polymer. CO_2_ lasers have also been used to heat the injected resin [44].

Ma et al. have thoroughly investigated the injection molding of PMMA-based microfluidic devices using a horizontal single screw injection molding machine capable of performing each injection cycle in 45 s (Figure 1c) [45]. They set the injection pressure at 120 MPa and the speed ranged between 200 mm/s to 400 mm/s. The injection was performed at 60 °C using an oil mold temperature controller. In another study, Kim et al. applied 5.5 MPa injection pressure and clamping force of 130 tons to create PS microfluidic channels at 220 °C in 15 s and used the device for single cell analysis [46]. Using the same injection molding parameters, Ko et al. fabricated an open circular microfluidic chip made of PS for ocular angiogenesis applications [47]. Injection molding was also adopted in Viehrig et al.’s work to form nanocones in COC through a nickel master mold (Figure 1d) [48]. The device was employed for SERS sensing applications.

In general, hot embossing and injection molding are more appropriate for medium-cost mass production through replication methods and can be implemented for the manufacturing of complex channel designs. Moreover, the quality of surface finish in these methods is superior compared to other methods, such as laser machining, micro-milling, and 3D printing. Injection molding is a very rapid method allowing for large-volume production. Hot embossing, in comparison, has an average production rate, but it requires less expensive tools and infrastructure. The primary disadvantage of injection molding pertains to limitations when fabricating microchannels with large footprints, whereas in hot embossing, large area machining is possible. It is worth mentioning that the polarity of the thermoplastic polymers affects their meltability. High polar polymers are very difficult to melt in their pure form due to their strong interchain forces [49]. Moreover, polar thermoplastics are not quite permeable to oxygen and carbon dioxide, which could be problematic in cell culture applications. Polar thermoplastics also have poor water barrier properties, which can lead to changes in local concentrations when implemented within applications that use water-based buffers and liquids. A comparison between the polarities of different thermoplastic polymers can be found elsewhere [50]. 

### 2.3. Master Mold Fabrication

The master molds used for the fabrication of thermoplastic polymers in both hot embossing and injection molding are usually fabricated by photolithography [17,18,24,51]. Nevertheless, several other fabrication methods, such as e-beam writing, electroforming, micro-milling, and electro discharge machining, laser machining, ion machining, additive manufacturing, and ultrasonic machining, are applicable for master mold production [35], as long as the master mold can withstand the high pressures and temperatures used in the hot embossing or micro-injection techniques. High-precision nickel molds [19,20,25,52], micro-milled aluminum [46,47,48], and Zr-based bulk metallic glass mold [36] are other master molds used in literature. For instance, a negative master mold was produced in Müller et al.’s work via electroplating Ni on a 3D printed master mold [52]. The negative master mold was then used for creating COC microchannels through injection molding. Hupert et al. used a high-precision micro-milling machine, which had positional and repetition accuracy of ± 1 µm, a laser measuring system and an optical microscope to create microstructures on a brass plate to be employed as a mold for the hot embossing of PMMA substrates [53]. In another interesting study, Perrone et al. used micro-second pulsed CO_2_ laser-based ablation to create microstructures on quartz [54]. The fabricated mold was then utilized to from hundreds of micron-sized pillars on COC substrates through hot embossing method. The final microfluidic device showed great potentialities in 3D cell culturing and organs-on-chip applications.

Micro-milling of brass templates has also been performed in other studies to create master molds for hot embossing microchannels in PMMA, PC, and COC substrates [55,56]. PDMS has also been utilized as a master mold for the fabrication of thermoplastic polymers (Figure 1e) [26]. In Chantiwas et al.’s study, PDMS master molds were prepared by casting PDMS at a base: curing agent ratio of 10:1 (*w:w*) in PMMA replicated micro/nano channels [55]. After curing the PDMS and peeling it off, it was used to hot emboss other PMMA substrates under a pressure of 0.16 MPa at 155 °C for 30 min. Schneider et al. generated a negative PDMS mold by casting PDMS on an SU-8 coated silicon wafer. They used this negative PDMS mold to produce an epoxy-based master mold for creating microfluidic channels made of TPE/PC, using hot embossing approach [31].

### 2.4. Laser Ablation

**Figure 2:**
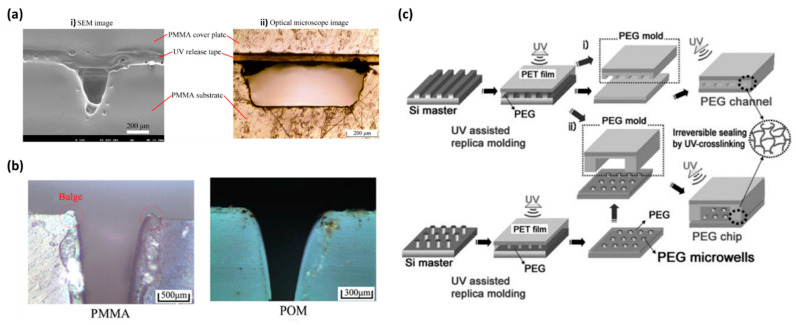
(**a**) (**i**) SEM image of PMMA microchannel’s cross-section fabricated by CO_2_ laser ablation (**ii**) Optical image of PMMA microchannel’s cross-sectional fabricated by micro-milling technique. Reproduced with permission [70] 2022, Springer. (**b**) Optical images of PMMA and POM microchannels’ cross-sections fabricated by laser ablation. Reproduced with permission [72] 2022, Springer. (**c**) Fabrication of PEG layers containing microchannels or microwells features and bonding to flat PET or PEG layers through UV polymerization technique. Reproduced with permission [97] 2022, Royal Society of Chemistry.

Another technique used for fabrication of the thermoplastic polymers with microfluidic channel cavities is laser ablation, which is applicable to many polymers, such as PC, COC/COP, PMMA, PS, nitrocellulose, polyethylene terephthalate (PET), PETE, and Teflon [57,58,59,60,61,62,63,64,65,66]. In this method, short laser pulses in the ultraviolet (UV) region (~200 nm wavelength) breaks the polymer chains. The decomposed polymer fragments, such as CO_2_ and CO gas, and polymer molecules are subsequently ejected due to the induced shock waves leaving photo-ablated cavities [60]. Patterning the microchannel arrays can be conducted by using photo masks in the process resulting in straight vertical walls without any significant thermal damage. It should be noted that laser ablation in the UV wavelength cannot be used for thermoplastic polymers, such as COC/COP due to their low UV absorption. Thus, infrared lasers, such as CO_2_ or Nd:YAG laser systems, should be adopted for microchannel formation. Namely, Liu et al. fabricated a COP-based microfluidic channel via CO_2_ laser ablation using pulse mode at a maximum frequency of 1 kHz, whereas a Gaussian-like profile was left on the surface of the COP plate as the COP melted, decomposed, and evaporated. They concluded that the main parameters affecting the profile of the microchannel included the power and scan speed of the laser, as well as the focusing accuracy of the laser and the mechanical transmission system [64]. CO_2_ laser ablation was also adopted to pattern PC and polylactic acid (PLA) sheets with the desired micro-features [57,67] and to create microchannel arrays in PET foils [68]. Laser ablation is also possible via desktop CO_2_-free laser cutters to create the sheets and membranes with the desired geometries [66]. Commercially available laser systems are flexible approaches for rapid redesign of channel geometries and are usually cheaper than some other techniques, such as injection molding, which requires metal molds or photolithography—a process that needs to be conducted in a cleanroom. The main drawbacks of laser ablation technique are the poor quality of the surface finish and its incapability for the fabrication of complex microchannel designs [69]. Further, the cut profile in conventional laser cutters is only limited to Gaussian-shaped profile or through cuts (Figure 2a) [70]. Formation of bulge along the scan route is another common problem associated with laser cutting technique [71]. Chai et al. showed that high thermal resistant thermoplastic polymers, such as polyformaldehyde (POM), can be CO_2_ laser cut without formation of bulges and carbide residue, and the channel depth and width are easily adjustable by changing the scan speed and laser energy (Figure 2b) [72]. Covering PMMA substrates by photoresist or PDMS is another way to tackle the bulge formation [73].

### 2.5. Additive Manufacturing

Nowadays, additive manufacturing of thermoplastic polymers has produced great interest in microfluidics due to its short fabrication cycle time. However, the resolution of 3D printed microchannels, mechanical properties, as well as the optical quality of the surface finish is not as good as the other techniques [74]. Additive manufacturing is a 3D digital manufacturing process that involves the fabrication of small batches of 3D parts layer-by-layer under accurate digital control, specifically used for applications that demand high-throughput production. Commonly used 3D printing methods include two photon polymerization, fused deposition modeling (FDM), selective selective laser sintering, stereolithography, laminated object manufacturing, and inkjet 3D printing [75]. A superior advantage of 3D printing is its ability to form three-dimensional structures with intricate and complex features with fewer space requirements in a single step from a digital model [74,76]. For 3D printing of thermoplastic polymers the most commonly used extrusion-based methods include FDM and inkjet printing, which employ materials, such as acrylonitrile butadiene styrene (ABS), polypropylene, polyamide, PLA, COC, PET, PS, and acrylate-based polymers [74,76,77,78,79,80,81,82,83,84,85]. Such methods offer several advantages, such as simplicity, low cost, less waste, usually high speed, and elimination of the need for bonding steps in some cases. FDM involves the extrusion of a heated thermoplastic material from a motor-driven nozzle head followed immediately by spontaneous cooling to harden the material. Thermoplastic filaments are the main printing material used in FDM, however by modifying the extruding nozzle other materials, such as powder or liquid thermoplastics, can also be used. FDM provides the most inexpensive and highly biocompatible productions due to a wide variety of cheap and biocompatible thermoplastics [76,78,79]. Despite its popularity in recent years, it still exhibits limitations when used in microfluidics, such as lack of structural integrity between the layers and weak bonding properties, since the adjacent layers are not well fused as the extruded material immediately hardens [75]. Recent efforts aimed to improve the intra-layer bonding strength of printed objects involve gamma-irradiation post printing [86], as well as employing thermally reversible Diels-Alder reaction to form covalent interactions upon cooling [76]. McAlpine’s group successfully fabricated a multi-scale biomimetic nervous-system-on-a-chip device to study viral infection in the nervous system. Using micro-extrusion FDM printing strategies, they created microchannels and compartmented chambers for the co-culture of neurons, glia, and epithelial cells using a custom FDM printer [87]. Dolomite microfluidics recently developed the first FDM printer to create completely sealed 3D microfluidic devices. Using COC, the printer creates leak-free, closed and impermeable microchannels [77]. 

Typically, additive manufacturing as well as micro-milling and laser ablation are more suitable for fast prototyping of thermoplastic microfluidics. Today, extensive research is being performed to advance the various 3D printing methods of thermoplastic-based microfluidics as an alternative for conventional manufacturing methods. Thus, recent works are mainly focused on the advancement of resolution, precision, optical characteristics, and more biocompatible structures [77,78]. For instance, exploiting the conductive properties of polyionic thermoplastic-elastomers and advancing the inks used in inkjet 3D printing holds great potential in developing more advanced and high-resolution microfluidics. In the near future, we anticipate the use of robots to automatically integrate electrodes, sensors, and actuators during printing of microfluidics, evolving the 2D microfluidic chips to 3D cubes [77,79]. Additive manufacturing of thermoplastic polymers is deeply discussed elsewhere [75,88].

### 2.6. Other Methods

Milling [89,90,91,92,93,94] and UV curing (specially for PEG) [95,96,97,98] are other methods to form the microchannels in thermoplastic polymers. Micro-milling of thermoplastic polymers can be incorporated for the fabrication of complex microchannel profiles with small or large surface areas. The production rate of this process is quite rapid, and it allows for instant changes in the channel design in the production line. Moreover, unlike laser ablation, it can create microchannels with nearly rectangular cross-sections. Nevertheless, the quality of the surface finish is not good in this process. In order to decrease the surface roughness and regain transparency after milling COC substrates, Bruijns et al. exposed the COC substrates to cyclohexane vapor at 60 °C for 1 min [93]. Micro-milling of PMMA substrates was demonstrated in a study done by Tomecka et al. [99]. They used a 500 µm and a 100 µm milling drums rotated at a speed of 12,000 rpm to create convex structures and oval-shape holes inside the convex structures, respectively. UV curing of low molecular weight polyethylene glycol (PEG) monomers, such as PEG dimethacrylate and PEG diacrylate (PEG-DA) on a silicon mold with the microchannels arrays or pillars, can be performed to fabricate PEG microchannels and porous PEG membranes, respectively (Figure 2c) [97]. During casting, a PET layer modified with urethane groups could be placed on top as the supporting layer to adhere to the acrylate-containing PEG monomers. As the supporting layer, it is also possible to use glass slides treated with phosphoric acrylate or acrylic acid dissolved in propylene glycol monomethyl ether acetate to bind to PEG [97]. In another study, Liu et al. first made an enclosed mold comprising a bottom silicon layer with the channel’s array, middle PDMS spacers, and a top glass slide [95]. Afterwards, PEG-functionalized monomer solution containing 85% PEG-DA, 12% poly(ethylene glycol) methyl ether methacrylate (PEG-MEMA), 3% methyl methacrylate, and 2,2′-dimethoxy-2-phenylacetophenone (DMPA) was injected onto the mold and cured for 16 s under UV. Tian et al. also UV cured PEG-DA on a PDMS mold using 1% photoinitiator Irgacure 2959 to form the microfluidic design [98]. UV lithography has also been used to create micro-geometries in PMMA substrates [100,101]. This process involves UV exposure of the PMMA substrate with a photoresist through a mask and development of the photoresist, coating a thick layer of X-ray absorber to the exposed areas to create an X-ray mask, emitting X-ray to form the desired channels, and finally removing the X-ray mask form the substrate.

Chandrasekaran et al. presented a new thermal scribing method to rapidly prototype thermoplastic microfluidic devices [102]. In this technique, a heating pen was incorporated into a commercially available craft cutter machine. The induced heat in the pen could locally raise the temperature of the thermoplastic polymer above the glass transition temperature and precisely pattern the layer with the desired geometry.

Another interesting way to fabricate microfluidic features is via the use of dry films, which were originally developed for printed circuit boards [103,104,105,106]. This technique is usually compared with photolithography—used for fabrication of SU-8 layers, or soft lithography, which is used to prepare molds for casting polymers, such as PDMS. While photolithography is an expensive method that needs cleanroom facilities and expert technicians, the fabrication of microchannels via dry films is a simple cleanroom-free approach that can provide comparable resolution and precision to SU-8 photolithography. Dry films resists (DFR) in different series, such as Ordyl, SUEX, and ADEX, TMMF S as well as SU-8 based DFR are commercially available in different thicknesses. DFRs can be laminated on a variety of different thermoplastic substrates or other types of materials via a simple office laminator. Subsequently, the layer is exposed to UV light through a photomask with the desired features and baked on a hotplate for a short period of time. Afterwards, the layer is immersed in a developer solution to form the cavities. The process of lamination and UV treatment can be performed multiple times to acquire microchannels with different heights or multiple layers (3D microfluidics). The microchannels can become hydrophilic through plasma treatment or polyvinyl alcohol if needed [106]. DFRs have also been utilized as a sealing layer in microfluidics made by injection molding. Moreover, researchers have used DFRs in fabrication of molds for hot embossing and PDMS casting processes [107,108,109]. The most common techniques for forming microfluidic channels are summarized in Table 1.

## 3. Bonding

Unlike hybrid PDMS/glass microfluidic devices, where surface hydroxylation via oxygen plasma treatment, ultraviolet-ozone (UVO) treatment, or corona discharge method is the main bonding technique, use of such systems for thermoplastic polymers usually lead to very weak bonding. Moreover, exposure of thermoplastic to UV, ozone or oxygen plasma could produce cytotoxic by-products such as hydrogen peroxide, which is troublesome for many biomedical applications and cell studies [111]. The most commonly reported techniques to irreversibly bond thermoplastic polymers together and form enclosed microfluidic devices include thermal fusion bonding [17,18,20,21,22,24,58,89,112], solvent bonding [19,25,26,32,59,113], and chemical bonding [114,115] (Table 2). Some recent advances in thermoplastic microfluidic bonding have also been discussed in another review paper published by Giri et al. [15]. 

### 3.1. Thermal Fusion Bonding

**Figure 3:**
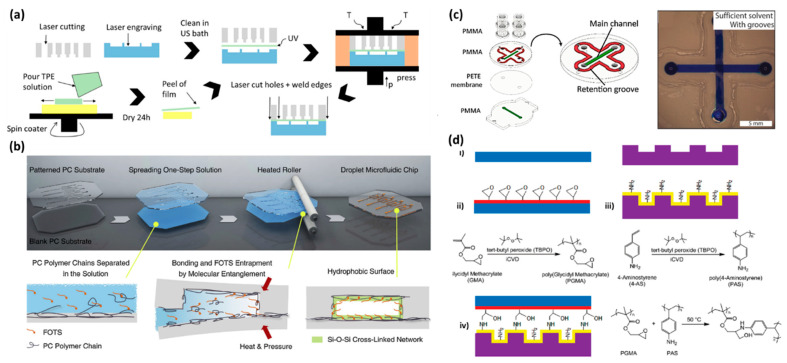
(**a**) Fabrication process of laser cut PMMA microchannels (pneumatic channels in gray and a fluidic channel in blue) sandwiching a TPE film. The device was bonded via UV assisted thermal fusion bonding. Reproduced with permission [30] 2022, MDPI. (**b**) Solvent bonding of PC microfluidic channels. FOTS molecules are entangled with the polymer chains and hydrolysed. Reproduced with permission [25] 2022, Elsevier. (**c**) Retention grooves embedded in microfluidic design to promote the solving bonding process in a PMMA-based microfluidic device. Reproduced with permission [66] 2022, Elsevier. (**d**) Chemical bonding of thermoplastic layers via iCVD polymerization of poly(glycidyl methacrylate) (PGMA) and poly(4-aminostyrene) (PAS). (**i**–**iv**) The induced chemical groups on the surface at each step. Reproduced with permission [115] 2022, ACS publications.

In thermal fusion bonding, the polymeric layers are heated above their glass transition temperatures, while the layers are pressed together using a hydraulic press or vacuum thermocompressor [89]. Compressing the substrates together using rollers at hot temperatures is also performed in thermal lamination techniques [61,62,96]. The print, cut, and laminate (PCL) approach has been shown to be an effective and inexpensive way to fabricate thermoplastic microfluidics made of polyester or other such polymers [110,116]. COC and PMMA microfluidic devices are widely fabricated using thermal fusion bonding method [20,24,36,58]. For example, thermal fusion bonding at 145 °C under 35 kPa was done to bond COC layers [20]. Moreover, PC-PC bonding was performed through thermal bonding at 143 °C and under 0.9 MPa pressure for 2 min [17]. Exposure of thermoplastic polymers to UV light before thermal fusion bonding can reduce the bonding temperature to below the T_g_ as a short-wavelength UV light is able to break polymer chains at the surface of the polymer [117]. Roy et al. UV grafted different monomers of acrylic acid (AAc), acrylamide (AAm), 2-hydroxyethyl methacrylate (HEMA), and N-vinylpyrrolidone (NVP) on a COC substrate using BP photoinitiator to indicate the best biocompatibility and ability to bond to another COC substrate at low temperature [36]. All the grafted coatings resulted in strong COC thermal fusion bonding at temperatures below the T_g_ of COC. UV light was also incorporated in Ongaro et al.’s work to bond PLA substrates together at a lower temperature. In their protocol, after UV exposure for 45 s, the substrates were placed in contact at 50 °C for 10 min to complete the bonding [67]. In Busek et al.’s study, 0.6 J/cm^2^ was demonstrated as the optimum UV dose for binding PMMA to PMMA as well as PMMA to TPE at 84 °C and 70 °C, respectively (Figure 3a) [30]. Exposing COP to UVO_3_ for 20 min followed by a thermal fusion bonding step has also resulted in good strength bonding between two COP layers [65]. Oxygen plasma treatment technique prior to thermal fusion bonding is also an alternative approach to reduce the thermal bonding temperature and enhance the bonding strength [118,119].

Transparency of the microfluidic device, resolution and precision of the microchannels, and bonding strength are important parameters that should be taken into consideration when an appropriate bonding method is selected. Thermal fusion bonding is a very fast technique to bond thermoplastic layers. However, it requires high pressure and temperature, which hinders its potential for the low-cost mass production of microfluidic devices. Deformation of channels after thermal diffusion bonding is also very plausible as this process is usually performed at temperature above the T_g_. Furthermore, in this process, the layers cannot be bio-functionalized prior to bonding as the high temperature used in this method would denature immobilized proteins and eliminate functional groups. Thus, the functionalization process should be performed after channel bonding, which can be more challenging considering the limited access to the inner surface of the channel after bonding. That said, perfusion of certain chemical reagents and crosslinkers followed by proper incubation and wash can help with functionalization of enclosed channels in a wet-chemistry manner [11,120,121]. 

### 3.2. Solvent Bonding

In this method, the applied solvent diffuses across the polymer interface and dissolves the polymer chains making them mobile. The polymer substrates are compressed and as the solvent evaporates, the induced mobile chains are entangled in each other at the interface and create a strong bonding force [25]. In order to avoid deformation of the polymer by the solvent, it is carried out at very low solvent concentrations, which increases the required bonding time [122]. Solvent bonding is usually stronger when it is used for bonding layers with the same material combinations and when it is integrated with thermal bonding [65].

For COC substrates, it is known that polar organic solvents cannot dissolve the polymer, while non-polar organic solvents, such as hydrocarbons, can dissolve it well [123]. As an example, in order to bond COC channels to a COC flat layer, the flat layer could be exposed to saturated methylcyclohexane vapor for 15 s at 30 °C followed by immediate contact to the COC channels at 85 °C for 15 min under 1 MPa pressure [19]. In another study, a COC lid was immersed in 15% decalin diluted in ethanol for 5 min to plasticize the COC. The layer was brought into contact with another COC layer with 1.7 MPa pressure at a temperature below the glass transition temperature [26]. Bruijns et al. exposed the COC substrates to a solution containing 40 vol% cyclohexane and 60 vol% acetone for 2 min [93]. Afterwards, the substrates were bonded together at room temperature under a weight of 2 kg.

PC-PC bonding has also been studied via solvent bonding using an acetone and n-pentane mixture (Figure 3b) [25]. The process initially involved immersing two PC substrates, one with patterned microfluidic channels and one without, into a solvent containing acetone, n-pentane, and 1H,1H,2H,2H-perfluorooctyl trichlorosilane (FOTS) [25]. The n-pentane solution acts as a sacrificial solvent and evaporates faster, leaving temporary high concentrations of the acetone for dissolving the substrate. The substrates were inserted into a heat roller with a pressure of 0.1 MPa, which results in the mobilized polymeric chains being entangled. The use of FOTS provides hydrophobicity after the bonding, which is not normally achievable as the surface energy of the substrates are usually increased in the bonding process to obtain better adhesion. Mirgissa et al. placed PC substrates at a certain distance from a chloroform pool to create surface swollen regions containing mobile polymeric chains for solvent bonding [57]. The solvent bonding was followed by thermal bonding at 125 °C to ensure the robustness of the bonding. They claimed that utilization of chloroform facilitated the thermal bonding process, which is normally conducted at higher temperatures and pressures for longer periods of time compared to their protocol.

PMMA-PMMA bonding has also been done by a UV and solvent assisted method, where the substrates were first soaked in ethanol and then aligned together and exposed to UV for 20 min [59]. Ethanol as a non-toxic solvent can dissolve PMMA and form acrylate monomers which diffuse across the interface of two layers and results in strong bonding upon the exposure of UV light. The device needed to be kept at 120 °C for 2 h under 1 kg weight [59]. Doung et al. applied the same method to make a hybrid PLA/PMMA microfluidic device [124]. They used a spin coater to uniformly spread ethanol onto the layers to prevent bubble formation. The bonding was performed at a low temperature of 55 °C. In another study, Doung et al. sprayed ethanol onto PMMA and ABS layers for bonding [83]. After bringing the layers in contact, the device was put under UV to generate the temperature (55 °C to 60 °C) needed for dissolving the surface layer of PMMA. They also performed a post-heat treatment to release the residual stress caused after UV exposure. In order to enhance the efficiency of solvent bonding method, grooves were embedded in Persson et al.’s design to retain solvent in the vicinity of microchannels. Using SciGrip 4 as the solvent, they managed to create strong bonding between PMMA microchannels sandwiching a PETE membrane (Figure 3c) [66]. 

Solvent bonding is usually not compatible with pre-functionalization as well, since normally both top and bottom thermoplastic layers should be treated with the solvent, which can eliminate the functional groups and denature the immobilized biomolecules. This method is achievable without denaturing pre-immobilized biomolecules only if the lid polymer layer (without the immobilized biomolecules) is in contact with the solvent. Moreover, the solvent bonding should be done at room temperature to maintain the functionality of the biomolecules, and the transparency of the layers should not deteriorate. PMMA-PMMA bonding at room temperature could be done using ethyl acetate and isopropanol (35:65% *v/v*) together with the lamination technique [32]. Furthermore, Keller et al. used 35% cyclohexane in acetone to dissolve a COC polymer layer causing it to become “tacky” [113]. They chose a non-penetrating polar acetone solvent to inhibit the excessive diffusion of the non-polar cyclohexane solvent into COC, which can result in bubble formation and lack of transparency. In the proposed protocol the COC with an array of pre-immobilized biotin was bonded with another COC lid layer that had become tacky. They proved that the bonding technique does not denature the biotin compared to thermal fusion bonding.

In general, solvent bonding is a longer process than thermal fusion bonding, but it does not require high temperatures, which could potentially reduce the fabrication cost. In solvent bonding, overexposure of the substrates to the solvent can result in deformation of the microfluidic channels and solvent residue could adversely affect the functionality of immobilized biomolecules. Therefore, the exposure time and concentration of the solvent are important factors in this method.

### 3.3. Chemical Bonding

Chemical bonding involves equipping the polymeric layers with functional groups and causing covalent reactions between these groups at the interface when the layers are brought into contact. Silanization in particular was used in Pečar et al.’s work to bond thermoplastic substrates, such as PC, ABS, and PMMA to PDMS microchannels [125]. The oxygen plasma treated thermoplastic substrates were coated with either a 5% 3-aminopropyltriethoxysilane (APTES) or a poly [dimethyl siloxane-co-(3-aminopropyl) methyl siloxane (Amine-PDMS). After proper heat treatment, the amine-functionalized thermoplastic substrates together with PDMS were again oxygen plasma treated and brought into contact using methanol as an aligning medium, followed by an additional curing at 80 °C for 1 h. Similarly, Sivakumar et al. chemically modified the surface of PDMS using anhydride silane and amino silane reagents, resulting in a permanent bond with PET at room temperature via the formation of a stable succinimide group, without the requirement of additional pressure to initiate bonding [126]. The hybrid PDMS-PET microfluidic channel can thus be used in high-pressure experiments, such as the separation of blood and plasma [126]. Bis-[3- (trimethoxysilyl)-propyl]-amine (Bisamino Silane) has also been utilized to bind PC/TPE-hybrid layers to different substrates, such as TPE, COC, PS, PC, and glass [31].

APTES treatment is also a common strategy for irreversible bonding of nano porous membranes, such as PC, polyethersulfone (PES), and PETE, to different substrates for organs-on-chips applications [65,127]. In this protocol, subsequent to O_2_ plasma activation, the membrane is submerged in a 5% APTES solution (diluted in DI water) at 80 ℃ for 20 min. Press the APTES treated membrane against a O_2_ plasma activated layer leads to an irreversible bonding between the layers. It is worth mentioning that the corona discharge method is not recommended for hydroxylation of membranes as it could damage the homogeneity of the membrane [127]. 

It has been shown that although silanization is an effective way to bond COC to PDMS, it is not generally applicable for COC-COC bonding [26]. The CVD polymerization of 4-aminomethyl [2,2]paracyclophane and 4-formyl[2,2]paracyclophane approach introduced by Chen et al. could also be a potential way for chemical bonding of thermoplastic polymers [128]. The CVD treatment involved sublimation of the precursors in vacuum and condensation of the precursors on the substrates at 15 °C. The induced poly(4-aminomethyl-p-xylylene)-co-(p-xylylene) (polymer 1) and poly(4-formyl-p-xylylene-co-p-xylylene) (polymer 2) coatings on the two layers of the microfluidic device could covalently bind to each other through the reaction between the amine groups of polymer 1 and aldehyde groups of polymer 2. To accomplish the covalent bonding, the substrates were placed in touch at 140 °C for 3 h. Use of initiated chemical vapor deposition (iCVD) is another way for chemical bonding polymers [114,115]. iCVD is a solvent-free process where a monomer together with an initiator are simultaneously introduced to the substrate at gas phase. A heating filament is used in the chamber to thermally radicalize the initiator at 150–300 °C [129]. The radicals react with the adsorbed monomer on the cool substrate and initiate polymerization. As iCVD is solvent-free and the polymer is maintained at (15–40 °C), the process does not generate delamination, swelling, shrinkage, or wrinkling on the membrane or sensitive polymers [129]. Im et al. employed this technique for chemical bonding of different polymers, such as PDMS, PS petri dishes, PET, PC, and poly(tetrafluoro ethylene) (PTFE) [114]. In the proposed protocol, allylamine plasma treatment was done to create polyallylamine (PAAm) on one layer. Poly(glycidyl methacrylate) (PGMA) formed another layer via iCVD using tertbutyl peroxide as the initiator and glycidyl methacrylate as the monomer. Oxygen plasma treatment prior to the coatings promoted adhesion for polymers with low surface energies, such as PTFE. After the coatings, the substrates were brought into contact under 0.1 bar pressure at 70 °C. The layers could covalently bind owing to the ring opening reaction between the epoxy and amine terminates.

The iCVD method is also used in a work done by Xu et al. [115]. Here, tert-Butyl peroxide was used as the initiator and 4-Aminostyrene (4-AS) as well as glycidyl methacrylate (GMA) as the monomers to create poly(glycidyl methacrylate) (PGMA) and poly(4-aminostyrene) (PAS) coatings, respectively. The process was applied on different substrates including PDMS, PC, PET, polyethylene (PE), polyacrylate (PA), and COC. Before iCVD, the substrates were oxygen plasma treated for better adhesion. PGMA and PAS films were polymerized on the flat and channelled layers, respectively. The layers could covalently bind without any need for high pressure or temperature. Moreover, the remaining functional groups inside the sealed channel could be further incorporated in covalent immobilization of biomolecules. They demonstrated this via the attachment of conjugated quantum dots via DCC chemistry (Figure 3d).

In comparison to thermal fusion and solvent bonding, chemical bonding allows for simultaneous functionalization of the inner surface of microchannels as the reactive chemical groups induced for bonding the two layers can also be incorporated for further covalent attachment of biomolecules. The other advantage of this method is the superior bonding strength as a result of covalent bonding of layers. Moreover, it is possible to conduct the bonding at low temperatures and pressures, which preserves the precision of the channels’ geometry better than thermal and solvent bonding [115]. The disadvantage of this method is the many steps involved for chemical treatment of the layers, which makes it troublesome for commercialization.

### 3.4. Ultrasonic Welding

**Figure 4:**
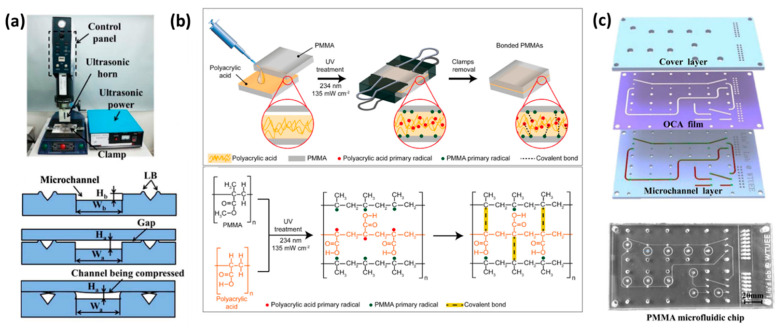
(**a**) Ultrasonic welding apparatus. In the microchannel design, the laser ablated grooves and bulges can create strong bonding and secure the melted polymer during the ultrasonic welding. Reproduced with permission [130] 2022, Elsevier. (**b**) Bonding PMMA layers through UV-curable PAA adhesive layer. Reproduced with permission [94] 2022, Elsevier. (**c**) Thermal bonding of PMMA layers using an optically clear adhesive (OCA) film. Reproduced with permission [63] 2022, Springer.

Ultrasonic vibration can also be utilized as a heating source, which significantly expedites the bonding process [33]. This process offers high bonding strength in a very short time, making it a good strategy for rapid prototyping. By controlling the pressure, power, and time, the bonding process can be optimized. Ultrasonic welding enables us to locally bond specific regions of the microfluidic layers. Thus, unlike conventional thermal bonding where the temperature of the entire chip is kept above the glass transition temperature, which can denature the preloaded biomolecules or possibly deform the channels, here, the generated heat energy is focused on energy directors (ED) at the specific regions that need to be bonded. The energy directors are essentially convex structures around the channels which are melted during ultrasonic vibration and stick the layers together. The melting flow, however, could leave a small gap between the bonding layers or could flow into the channels and clog the microchannels. In order to tackle this problem, Liang et al.’s incorporated a CO_2_ laser ablation system in the ultrasonic welding process [130]. Using the CO_2_ laser cutter, they formed grooves and laser bulges as the energy director. Therefore, during the welding process, the melted laser bulges could flow into the grooves and consequently prevent the gap and clogging issues (Figure 4a). Viehrig et al. also used laser ablation to engrave grooves on an aluminum-based master mold created by micro-milling technique [48]. The grooves were 50 µm in both depth and width and served as energy directors in ultrasonic welding. Ultrasonic welding can also be employed for bonding microfluidic connectors and luers to the device [131].

### 3.5. Laser Welding

Another approach to bond thermoplastic polymers is laser welding. In this process, the substrates are held in contact and a laser beam is transferred through the whole platform to reach and melt the materials at the interface for bonding. Similar to ultrasonic welding, this process also creates localized heating and can rapidly bond the layers together. However, the transparency of the substrates is an important matter in laser welding. The top layer should be sufficiently thin and transparent to the laser wavelength so that the laser does not lose the required energy for melting the interface, while it is passing through the top layer. On the other hand, the bottom layer should be absorbent to the laser beam so as to be able to heat the interface by absorbing the laser energy. To make the bottom layer opaque to the laser beam, usually carbon black (CB) pigments are used. As an example, Archerjee et al. investigated laser welding of PMMA to ABS via a diode laser at 809.4 nm wavelength [132]. In their study, the transparent PMMA was placed at top and the opaque ABS with 0.1 wt% of CB pigments was placed at the bottom. The laser power, area of the beam spot, and welding speed are the parameters that should be optimized in laser welding to achieve the best efficiency. Moreover, high clamping pressure and good surface finish of the substrates are required in order to eliminate any air gaps in between that may cause a weak bond. 0.40 wt% of CB was also melt blended in polystyrene in Juhl et al.’s study, allowing for laser bonding to another polystyrene layer [133]. The CB can also be added only at the interface between the two layers [134]. Nowadays, with advancements in laser beam sources, it is possible to generate lasers at the wavelengths of approximately 2 µm, namely, by using thulium fibre lasers. This avoids the necessity for an additional absorbent layer as thermoplastic polymers are already absorbent in this range. However, the heat affected zone in these wavelengths is more extended across the thickness of the layers due to the absorbance of laser energy by the thermoplastic polymer along the transmission. Thus, the thermal stress in this technique is normally unavoidable [135]. Pelsmaeker et al. have conducted a thorough investigation on laser welding of different thermoplastic polymers at a laser wavelength of 2 µm [136].

### 3.6. Adhesive Layers

Pressure sensitive double-sided adhesives, such as acrylic and silicone-based tapes, have frequently been used for microfluidic device bonding [52,65,68,72,137,138,139]. The adhesive tapes can simply be cut via a laser cutter or a vinyl cutting machine to form the geometry of the microchannels within the tape. Afterwards, the tapes are sandwiched between two flat thermoplastic layers under mild pressures to create an enclosed microfluidic device. Adhesive tapes can greatly expedite manufacturing process of multilayered microfluidic devices and do not deteriorate the transparency of the device [65]. Moreover, as adhesive taps are gas permeable, the intermediate adhesion layer can compensate the impermeable nature of thermoplastic layers, which is a critical factor for cell studies [138]. Another advantage of adhesive tapes is that the substrate can be functionalized and coated with the biomolecule of interest prior to bonding. It is important to consider the cytotoxicity and adsorption properties of the adhesive tapes when they are applied for cell culture and biosensing applications [52,140].

Sathish et al. used this strategy to bond a major outer membrane protein (MOMP)-functionalized PMMA substrate to another clear PMMA substrate [141]. In their design, the geometry of the microchannel was cut out in a double-sided tape via a CO_2_ laser cutter and subsequently the tape was sandwiched between the two flat PMMA substrates. Chai et al. incorporated a hydraulic thermal press to enhance the bonding strength of a POM microfluidic device bonded via a pressure sensitive adhesive layer [72]. Mylar tapes have also been laser cut to form the channels and then used for bonding layers made of PS [142,143]. Bonding properties of dry adhesive tapes for thermoplastic microfluidics are fully investigated in a work done by Tsao et al. [144].

UV-curable adhesives are another method for bonding of thermoplastic layers. In this process, the adhesion solution, such as poly(acrylic acid) (PAA) and Norland Optical Adhesives (NOA), is first spread on the bottom layer using spin coating, for instance, and subsequently the second thermoplastic layer is placed on top. The two parts are pressed together under UV irradiation for a few minutes [63,94,145]. Le et al. showed that the remaining PAA inside the channel can be used for collogen coating through electrostatic interactions, which is highly beneficial for cell culture and organs-on-chips applications (Figure 4b) [94]. It is also possible to partially UV cure the adhesive layer on a flat PDMS slap and then transfer it to another substrate through a stamp and stick process. After transferring the pre-cured adhesive layer, the second layer of the microfluidic channel is placed on top and the device is fully cured under UV light to obtain irreversible bonding [145]. UV curable release adhesives have also been used to achieve reversible bonding. In this method, after bonding the layers, the acrylate oligomers in the adhesive tape can be cross-linked under UV exposure and become rigid, thus losing the adhesive’s strength [70].

Liu et al. [63] showed a low temperature bonding method in which they utilized an optically clear adhesive (OCA) film to bind PMMA layers. The device was composed of a cover layer, OCA film, and microchannel layer (Figure 4c). The channel design was initially formed on all of the layers using standard laser ablation. The layers were laminated by a heat pressing at 45 °C for 600 s to achieve the most effective bonding area. Afterwards, they perfused a liquid optically clear adhesive (LOCA) into the channel to fill the crevices between the layers. The LOCA was then blown out by nitrogen gas and the thin LOCA coating remaining inside the channel was solidified under UV light for 2 h.

In order to bond microfluidic devices made of PEG, partially cured or uncured PEG is usually placed between the layers, which can then be fully photopolymerized under UV light [95,97,98]. Particularly, in Liu et al.’s work, after formation of the PEG microchannels, a lid PEG layer was injected in a mold and semi-cured [95]. The two PEG substrates were then brought into contact and fully cured via exposure to UV light for 5 s, which led to the formation of covalent bonds between methacryl and acryl residues in both PEG layers. The device was then immediately compressed under a 2.4 kg weight for 3–4 min to be flattened. The device was used for electrophoresis separations of fluorescein isothiocyanate-labeled protein and peptide samples without further modification owing to the adsorption-resistant nature of PEG. Kim et al. applied a slight physical pressure of ~10^3^ Pa during the UV curing to make conformal contact (Figure 2c) [97]. The UV exposure was performed for a few minutes to induce crosslinking at the interface creating an irreversible bond. More interestingly, Tian et al. utilized this method to sandwich a porous membrane functionalized with silane-(PEG)_5000_-NHS between PEG layers [98]. After covalent attachment of anti-*E. coli* or anti-*S. aureus* antibodies to the membrane through the reaction between NHS ester and amine groups of the antibodies, they utilized the device for electrochemical detection of food-borne pathogens.

Use of PDMS as a glue at the interface could also be an option for providing a strong bonding force [146]. In this case, a thin layer of uncured or semi-cured PDMS is introduced on the surface of one or both of the bonding layers and after bringing the layers into contact, the platform is heated to fully solidify the PDMS at the interface thereby fastening the layers to each other. Although PDMS glue and adhesive tapes can provide relatively high bonding strength, the resolution and accuracy of the microchannels are not as good as the other methods. Moreover, regarding the uncured or semi-cured PDMS, the leakage of PDMS into the channel and consequently changing the channel geometry or clogging the channel is a common problem. In addition, PDMS shrinks as the device cools, resulting in misalignment of the channels.

## 4. Conclusions and Future Trend

The use of thermoplastic polymers within microfluidic systems provides a tremendous opportunity for low-cost mass production of microfluidic devices. Replication methods, such as hot embossing or injection molding, as well as fast prototyping methods, including micro-milling and laser ablation, represent some of the inexpensive approaches that are compatible with thermoplastics—strategies that are normally not applicable to devices made of PDMS or silicon-based materials [5,6,155]. Recently, additive manufacturing and 3D printing techniques have gained more attention for their use in the rapid manufacturing of thermoplastic microfluidics—usually composed of PLA and ABS, due to technological advancements that have yielded effectiveness with complex designs and improved precision. Additive manufacturing of microfluidics is especially useful for the fabrication of innovative monolithic and heterogeneous microfluidics in which multiple layers of chips with different functionalities are stacked together to obtain a small 3D platform exhibiting superior performance. These systems have shown high applicability within complementary metal oxide semiconductor (CMOS)-integrated optical and electrical sensors, microfluidic cooling systems on 2.5D or 3D integrated circuits (ICs) and some other lab-on-a-chip applications [156,157,158].

## Figures and Tables

**Table 1:** Common approaches for forming microchannel.

Method	Experimental Procedure	Effective Parameters	Advantages	Disadvantages	Examples
Hot embossing	Heating polymer plates above Tg Pressing against a master mold Cooling down Detachment of the formed channels	Heating and cooling temperatures Pressure and time of embossing process	✓ High replication accuracy ✓ Capability for mass production especially by integration with roll-to-roll printing ✓ Good surface finish quality ✓ Allows for fabrication of complex microchannel designs ✓ Medium cost	✕ Average production rate ✕ Requires a master mold ✕ Difficulties in forming channels with high aspect ratios ✕ Possibility of breakage during the detachment step	PMMA [22,32,53,55,56] COP/COC [19,20,22,24,26,27,28,36,54,55,56] PC [17,18,33,55,56] PS [21] TPEs [29,30,31]
Injection molding	Melting polymer granules Injection into the mold cavity Cooling down Ejection	Melt temperature and mold temperature Heating and cooling rate Filling rate Packing time and pressure	✓ A rapid process especially by integration of rapid heating and cooling systems, such as conformal cooling and variotherm systems ✓ Tight tolerances and High reproducibility	✕ Requires expensive tools ✕ Incapable of producing channels with large footprints ✕ Requires a master mold	PMMA [45] PC [25] COC [36,48,52] PS [46,47]
Laser ablation	Decomposing polymer chains by irradiation of laser through a photo mask Ejection of the polymer fragments due to the induced shock waves Cooling down	Laser power, wavelength and frequency Scanning speed	✓ A relatively low-cost technique ✓ A rapid process ✓ Flexibility in on-the-fly modification of the design	✕ Poor surface finish quality ✕ Incapable of fabrication of complex microchannel designs ✕ Causes bulge formation ✕ Creates Gaussian-shaped cut profile	PMMA [58,59,63,65,66,69,70,71,73] PC [57,60] PS [60] PET [60,61,62,68,110] PETE [66] COP [64,65] PLA [67] POM [72]
Micro milling	Modeling the channel design Mounting the substrate on the machine and conduct the milling	Milling speed Spinning speed of milling drum Teeth location on milling drum	✓ Allows for fabrication of complex microchannel designs ✓ Rapid production rate ✓ Flexibility in on-the-fly modification of the design	✕ Poor surface finish quality ✕ Poor transparency ✕ High precision and surface smoothness make the process expensive	PMMA [89,92,94,99] PC [90] COC [91,93]
UV-curing	Injection of polymeric solution into/onto a mold Irradiation of UV for curing	Layer thickness UV exposure time and intensity	✓ Very high resolution ✓ Provides non-biofouling properties when PEG is used	✕ Only applicable to few polymers ✕ Requires master molds or photo masks ✕ A challenging approach for mass production	PEG-based [95,97,98] PMMA [100,101]
Thermal scribing	Modeling a CAD design Inducing channel geometry by a heating pen installed on a craft cutter machine	Heating temperature Cutting speed Proximity of the cutting pen to the plastic	✓ A rapid technique ✓ Inexpensive method ✓ Simplicity of the process	✕ A time-consuming process ✕ Suited for low volume prototyping	PS [102]
3D printing	Preparing a CAD model of the design Loading the design into slicing software Printing the design	Physical and chemical properties of the resin Printer resolution	✓ A low-cost method ✓ Relatively fast technique for small footprints ✓ Capability for fabricating complex channel designs ✓ Flexibility in quick modification of the design	✕ Poor surface finish quality and optical transparency ✕ Low resolution ✕ Time consuming process for large footprints	ABS [80,83,84,85] PLA [81,82,84,86] COC [77] Acrylate-based [86]
Dry films	Lamination of dry film resists on a thermoplastic substrate UV exposure through a photomask Baking on a hotplate Submerging in a developer solution	Thickness of the laminated film UV exposure time and intensity Baking temperature and time Developer type and immersion duration	✓ Unlike photolithography, it does not require cleanroom facilities	✕ Requires a photomask ✕ Multiple process cycles should be conducted to form channels with different heights	ADEX [103] SUEX [103,104,105] Ordyl [106]

**Table 2:** Common strategies for bonding microfluidic layers.

Method	Experimental Procedure	Effective Parameters	Advantages	Disadvantages	Examples
Thermal fusion bonding	Heating polymer layers above Tg Pressing the layers together	Temperature Pressure Bonding time	✓ A rapid technique especially with incorporation of rollers ✓ Very strong bonding strength	✕ An expensive method for mass production ✕ Chance of channel deformation ✕ Layers cannot be (bio)functionalized prior to bonding ✕ Limitations in producing channels with large footprints	PMMA [30,58,89,118,119] PET [61,62,110] COC [20,24,36,117] COP [65] PC [17] PLA [67] TPE [30,118] Dry films [96] Polyester [116]
Solvent bonding	Application of a proper solvent to create mobile chains at polymer’s surface Pressing the layers together Evaporation of the solvent	Choice of solvent Concentration of solvent Exposure time	✓ A rapid technique ✓ Low cost ✓ Good optical transparency ✓ Very strong bonding strength especially when it is integrated with thermal bonding	✕ Usually, not compatible with pre-(bio)functionalization of the channels ✕ Deformation of channels is plausible ✕ Solvent residue could be problematic	PMMA [32,59,66,83,124] COC [19,26,93,113,123] PC [25,57] PLA [124] ABS [83] PETE [66]
Chemical bonding	Functionalization of polymers with chemical reagents Placing the layers in contact with each other.	Depends on the functionalization strategy of interest	✓ The functional groups can further be used for immobilization of biomolecules and other entities ✓ Very strong bonding strength due to the induced covalent bonds ✓ Usually does not require high temperatures	✕ A complex procedure for commercialization ✕ A time-consuming procedure	PMMA [125] COC [26,31,115] TPE [31] PS [31,114] PC [31,65,114,115,125] PET [114,115,126] PES [127] PETE [127] ABS [125] PA [115] PE [115] PTFE [114]
Ultrasonic welding	Application of ultrasonic vibration to heat polymers Pressing the layers together	Ultrasonic power Time Pressure	✓ A very rapid technique ✓ An inexpensive technique suitable for mass production ✓ High bonding strength ✓ Allows for (bio)functionalization of the channels is possible prior to bonding	✕ Energy directors should be embedded close to channels ✕ Channel deformation, clogging, and gaps between the bonded layers are probable ✕ Limitations in channels heights due to the polymer shrinkage	PMMA [130,147,148,149] COC [48,150] PC [131,149] PET [131]
Laser welding	Pressing the polymeric substrates in contact Irradiation of a laser beam at the interface	Laser power Welding speed Polymer absorption rate to the laser	✓ A very rapid technique ✓ High bonding strength ✓ Allows for (bio)functionalization of the channels is possible prior to bonding	✕ One layer should be thin and transparent, and the other layer should be absorbent to the laser ✕ Requires high clamping pressure and good surface finish ✕ Causes thermal stress throughout the polymer	PMMA [132,151] COC [152] PC [153] TPE [153] PS [134] ABS [132]
Adhesive layers	Cutting the channel geometry on a double-sided adhesive layer and sandwiching it between two polymeric substrates Spreading a UV curable adhesive layer between two polymeric substrates and irradiating UV light Spreading uncured or semi-cured PDMS between two polymeric substrates and heating the polymers	Type of adhesive layer Pressure UV light intensity and duration (for UV curable adhesive layers) Temperature and time (for uncured PDMS method)	✓ A rapid technique ✓ The polymer transparency is preserved ✓ Provides gas permeability for cell culturing applications ✓ Allows for (bio)functionalization of the channels is possible prior to bonding	✕ Cytotoxicity and adsorption properties of the tapes could be problematic for biological applications ✕ Limitation in channel thickness for double-sided adhesive layers ✕ Not applicable to all polymers ✕ Channel deformation and clogging is probable when uncured PDMS and UV curable adhesive are used	PMMA [63,65,70,94,141,154] COC [52,137] PET [68] Polyester [72] PTFE [154] POM [72] PS [142,143] PEG [95,97,98] PC [146]

## Data Availability

Not applicable.
